# Proteomic Analysis of Cell Walls of Two Developmental Stages of Alfalfa Stems

**DOI:** 10.3389/fpls.2012.00279

**Published:** 2012-12-13

**Authors:** Julian C. Verdonk, Ronald D. Hatfield, Michael L. Sullivan

**Affiliations:** ^1^U.S. Dairy Forage Research Center, Agricultural Research Service, United States Department of AgricultureMadison, WI, USA

**Keywords:** alfalfa, cell wall protein, shotgun proteomics, cell wall digestibility, cell wall protein database

## Abstract

Cell walls are important for the growth and development of all plants. They are also valuable resources for feed and fiber, and more recently as a potential feedstock for bioenergy production. Cell wall proteins comprise only a fraction of the cell wall, but play important roles in establishing the walls and in the chemical interactions (e.g., crosslinking) of cell wall components. This crosslinking provides structure, but restricts digestibility of cell wall complex carbohydrates, limiting available energy in animal and bioenergy production systems. Manipulation of cell wall proteins could be a strategy to improve digestibility. An analysis of the cell wall proteome of apical alfalfa stems (less mature, more digestible) and basal alfalfa stems (more mature, less digestible) was conducted using a recently developed low-salt/density gradient method for the isolation of cell walls. Walls were subsequently subjected to a modified extraction utilizing EGTA to remove pectins, followed by a LiCl extraction to isolate more tightly bound proteins. Recovered proteins were identified using shotgun proteomics. We identified 272 proteins in the alfalfa stem cell wall proteome, 153 of which had not previously been identified in cell wall proteomic analyses. Nearly 70% of the identified proteins were predicted to be secreted, as would be expected for most cell wall proteins, an improvement over previously published studies using traditional cell wall isolation methods. A comparison of our and several other cell wall proteomic studies indicates little overlap in identified proteins among them, which may be largely due to differences in the tissues used as well as differences in experimental approach.

## Introduction

Cell walls are dynamic structures that undergo significant changes during plant growth and development. A key function of cell walls is to define the basic physical characteristics of the plant, much in the way walls of buildings control their overall shape and size. However, unlike a building, plant cell walls remain dynamic and ever changing. Only the vessel elements (part of the vascular system) become inert upon full development. The combination of various cell types leads to unique tissues that form the complete plant. Complex structural carbohydrates (cellulose, hemicellulose, and pectin) form the bulk of the materials that make up most cell walls. These raw materials are important sources of fiber and feed.

In agriculture, the cell wall has nutritional importance as an energy source for ruminant livestock. With increasing efforts to convert plant biomass to energy, conversion efficiency of complex carbohydrates to biofuels is critical to developing a sustainable bioenergy program. It is likely that many of the limitations to conversion efficiency (i.e., digestibility) are the same for animal and bioenergy production systems. Generally, as the plant develops and matures, a large portion of the stem biomass is cell wall, and during maturation interactions among cell wall components (by hydrogen bonding, ionic bonding with Ca^2+^ ions, covalent ester linkages, and van der Waals interactions, Buchanan et al., 2000) result in increased structural integrity. In alfalfa (*Medicago sativa*), whose stems typically constitute approximately 50% of harvested biomass (Hatfield, 1992), stem cell wall digestibility decreases rapidly with maturation. This decrease is probably due in large part to increased crosslinking of cell wall components (Grabber et al., 2002). Besides lignin, a polymer composed of phenylpropanoid units, it is unclear what other components have a role in crosslinking in alfalfa cell walls.

To improve cell wall properties with respect to animal and bioenergy production systems, much research has focused on the manipulation of lignin, whose crosslinking to other cell wall components is associated with limiting digestibility (Vanholme et al., 2008). Cell wall proteins (CWPs) comprise a smaller fraction of the cell wall than structural carbohydrates (about 10–20% by dry weight) and likely play regulatory, enzymatic, and structural roles in the cell wall (Burke et al., 1974; Cassab and Varner, 1988). The chemical interactions (e.g., crosslinking) between cell wall polymers is sometimes facilitated by a specific class of CWPs, referred to as structural proteins (SPs). Thus, CWPs with regulatory, enzymatic, and structural roles could also all be potential targets for modification in crop plants to improve cell wall properties, especially with respect to digestibility.

To gain information about the population of proteins present in the cell wall, several proteomic analyses have been carried out on cell cultures, seedlings, leaves, etiolated hypocotyls, protoplasts, and roots of arabidopsis (Robertson et al., 1997; Chivasa et al., 2002; Borderies et al., 2003; Borner et al., 2003; Feiz, 2004; Schultz et al., 2004; Boudart et al., 2005; Charmont et al., 2005; Kwon et al., 2005; Bayer et al., 2006; Minic et al., 2007; Irshad et al., 2008). Although arabidopsis is an excellent model system for many processes, it does not produce significant woody tissues, nor does it have a true stem. Therefore, it is unclear whether studies of arabidopsis alone can provide all the needed information on factors limiting digestibility. Although much of the available data is dominated by studies on arabidopsis, studies of the cell wall proteome of other plant species including tobacco (Dani et al., 2005; Millar et al., 2009), chickpea (Bhushan et al., 2006), maize (Zhu et al., 2007), and alfalfa (Watson et al., 2004) have also been carried out.

Proteomic analyses of the plant cell wall present special challenges. In particular, difficulties arise when isolating proteins from the complex structure of cellulose, hemicelluloses, pectins, lignin, and SPs that makes up the cell wall. The exposure of the acidic polysaccharide network of the wall to intracellular proteins can trap them via ionic interactions as contaminants (Boudart et al., 2005). Another problem is the lack of a surrounding membrane can result in the loss of protein during the isolation process (Feiz et al., 2006; Jamet et al., 2008). Finally, a portion of the proteome is a structural component of the cell wall connected by covalent bonds and other strong molecular interactions (Brady et al., 1996; Jamet et al., 2008) making those proteins difficult or impossible to isolate. In most previous proteomic studies, CWP isolations used a long sequential washing procedure of cell wall material, in a range of buffers and salts to remove the cytosolic proteins while keeping the CWPs intact. This was followed by the extraction of CWPs from the isolated cell wall material by a CaCl_2_ treatment to release proteins and a LiCl treatment to extract more tightly bound proteins from the wall matrix (Hills et al., 1975; Morrow and Jones, 1986; Melan and Cosgrove, 1988; Feiz et al., 2006). Several cell wall isolation methods were compared and evaluated by Feiz et al. (2006), who identified critical steps to prevent contamination of cell wall preparations with intracellular proteins. They introduced a method using low ionic strength buffer washes, density gradients, and no detergents for the initial cell wall isolation. This method substantially decreased the proportion of presumptive intracellular proteins (e.g., those targeted to intracellular compartments and other non-secreted proteins) compared with previously used wall isolation methods (Feiz et al., 2006).

Due to the agronomic importance of alfalfa, much would be gained by understanding the metabolic processes that lead to reduced cell wall digestibility. Transcriptomics approaches have led to an improved knowledge of the cell wall of *Medicago* species (Minic et al., 2009; Yang et al., 2010), but there has been only a single proteomics analysis of the cell wall of alfalfa carried out nearly a decade ago by sequencing proteins isolated from 2-D gels (Watson et al., 2004). Here, we present a new analysis of the cell wall proteome examining two maturities of alfalfa stem, apical stems (less mature, more digestible), and basal stems (more mature, less digestible). To reduce intracellular contamination, we used the cell wall isolation method of Feiz et al. (2006). A modified procedure utilizing EGTA to remove pectins and facilitate protein recovery was used to extract proteins from the wall material, and recovered proteins were analyzed by shotgun proteomics. Using this approach, 272 proteins were identified, including 153 not previously identified in cell wall proteomic analyses. This data set should prove useful in developing strategies to improve digestibility of this and other forage crops.

## Materials and Methods

### Plant material

Following 30 days of regrowth, alfalfa stems (*M. sativa* cv. Ledgendairy 5.0) were harvested on the 24th of August 2011 from a field at the US Dairy Forage Research Center Farm in Prairie du Sac, WI, USA. The plants were at 10–20% flowering.

### Cell wall isolation and protein extraction

Stems were divided into basal and apical sections by taking the bottom two-fifths as basal and the top two-fifths as apical. The samples were frozen in liquid nitrogen, ground in a Sample Prep 6870 Freezer Mill (SPEX Sampleprep, Metuchen, NJ, USA) and stored at −80°C. Cell wall isolation was carried out as described by Feiz et al. (2006). Briefly, for each stem sample 50 g of ground tissue were washed overnight in a 500 ml bottle with 200 ml 0.4 M sucrose in 5 mM Na acetate buffer, pH 4.6, on a rocking platform at 4°C. The next day, the slurry was transferred to four 50 ml tubes, and centrifuged at 1000×*g* for 15 min. The supernatant was saved, and the pellets were washed by resuspending in 100 ml total volume with 0.6 M sucrose in 5 mM Na acetate buffer, pH 4.6; rocking at 4°C for 30 min after consolidating the samples into two tubes; and centrifuging at 1000×*g* for 15 min. This washing procedure was repeated with 1.0 M sucrose in 5 mM Na acetate, pH 4.6 then twice with 5 mM Na acetate, pH 4.6. For the final wash, the material was transferred to three Oak Ridge 30 ml centrifuge tubes (Thermo Fisher Scientific, Waltham, MA, USA) to facilitate subsequent extraction of protein from the pelleted cell wall material. For the EGTA protein extraction, the pelleted residue was taken up in approximately 10 ml per tube of 50 mM EGTA in 5 mM Na acetate, pH 4.6, and shaken vigorously at 37°C for 1 h. The samples were centrifuged for 15 min at 10,000×*g*, to obtain the supernatant containing the EGTA protein isolate. The pellet was extracted two additional times in this manner, all supernatants were pooled as the EGTA protein fraction, and stored at 4°C. The pelleted residue from the final EGTA extraction was taken up in approximately 10 ml per tube of 3 M LiCl in 5 mM Na acetate, pH 4.6, and placed on a rocking platform at 4°C overnight. The samples were centrifuged for 15 min at 10,000×*g*, to obtain the supernatant containing the LiCl protein fraction. The pellet was extracted two additional times in this manner (except the second extraction was for 8 h) and the supernatants were pooled and stored at 4°C. The EGTA and LiCl fractions were concentrated by using Amicon Ultra-15, PLGC Ultracel-PL Membrane, 10 kDa columns (Cat# UFC901024, Millipore, Billerica, MA, USA), with the final volume between 200 and 500 μl. Final protein concentrations were determined with the Pierce 660 nm Protein Assay (Thermo Fisher Scientific).

### Protein preparation

**Figure 1:**
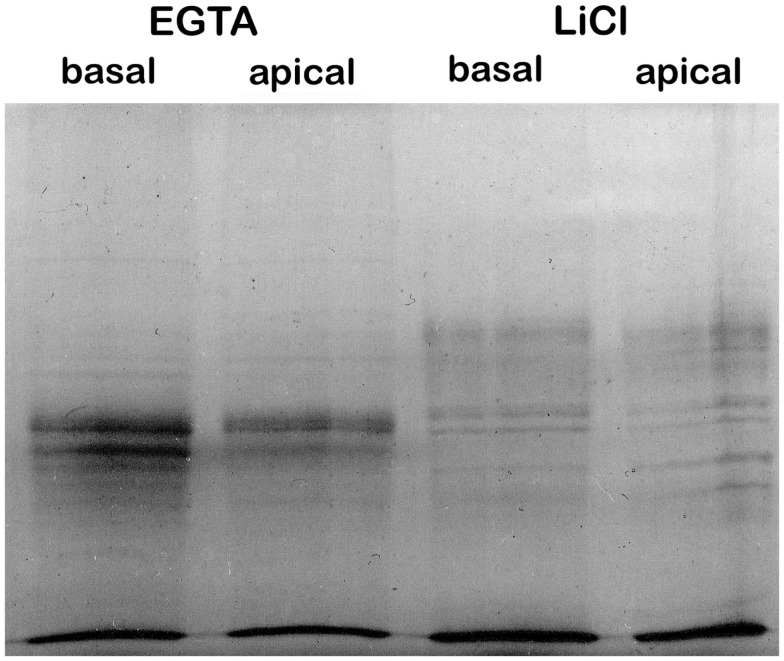
**Sequential EGTA-treatment and LiCl extraction of cell walls creates distinguishable protein fractions**.

The protein samples were concentrated by precipitation as described by Wessel and Flugge (1984). One hundred and fifty microliters of protein sample was mixed with 600 μl of methanol, 150 μl chloroform, and 450 μl of water in a micro centrifuge tube. The whole mixture was centrifuged for 5 min at 17,000×*g* at 4°C. The upper phase was removed, leaving the white, protein containing interphase intact, and 600 μl of methanol was added. The tube was centrifuged 5 min at 17,000×*g* at 4°C, the supernatant was removed and the protein pellet was dried. The pellet was resuspended in 50–100 μl SDS-PAGE sample buffer (0.3125 M Tris pH 6.8, 10% SDS, 50% glycerol, 0.025% Bromphenol blue, 25% 2-mercaptoethanol) and the protein concentration was again measured with the Pierce 660 nm Protein Assay (Thermo Fisher Scientific). Samples (2 μl corresponding to 5.5, 3.0, 5.5, and 5.0 μg of protein from basal EGTA, apical EGTA, basal LiCl, and apical LiCl, respectively) were resolved on a 12% SDS-PAGE gel using standard methodologies (Laemmli, 1970) to assess the quality of the protein samples (Figure 1). To prepare protein for proteomic analysis, 113 and 209 μg protein samples from EGTA and LiCl fractions, respectively, for both apical and basal stems were electrophoresed 1 cm into a 12% Tris-HEPES-SDS-PAGE gel (Pierce Precise Protein Gels, Cat# 25202, Thermo Fisher Scientific). Although proteomic analysis of ∼200 μg of protein would be optimal (D. Whitten, Michigan State University, personal communication), low yield of protein from the EGTA fraction necessitated using less. Analysis of less protein from the EGTA fractions was taken into account by the MaxQuant software which can correct for differential protein loads across experiments (Griffin et al., 2010; Cox et al., 2011). The gel was stained with R250 Coomassie [0.1% (w/v) Coomassie R250 dye in 50% (v/v) methanol 10% (v/v) acetic acid], and destained with 50% (v/v) methanol 10% (v/v) acetic acid. The protein containing gel for each sample was excised and stored in 50% (v/v) methanol 10% (v/v) acetic acid until used for proteomic analysis.

### Proteomic analysis

Gel pieces were sent to the Michigan State University Proteomics Facility^1^ for proteomic analysis as follows. Proteins contained within gel pieces were digested with trypsin in-gel and the resulting peptides extracted essentially as described by Shevchenko et al. (1996) and Nesvizhskii et al. (2003). The extracted peptides were resuspended to 20 μl in a solution of 2% (v/v) acetonitrile, 0.1% (v/v) trifluoroacetic acid in water. Peptides were resolved using a Water’s nanoAcquity UPLC system (Waters, Milford, MA, USA). Ten microlitre samples were loaded over 5 min onto a Waters Symmetry C18 peptide trap column (5, 180μm × 20 mm) in tandem with a Michrom MAGIC C18AQ column (3 μm, 200 Å, 100 μm × 150 mm, Bruker-Michrom, Auburn, CA, USA) at 4 μl/min in 2% (v/v) acetonitrile, 0.1% (v/v) formic acid in water. The bound peptides were eluted over 90 min at a flow rate of 1 μl/min with a gradient of 5–35% solvent B over 78 min; a gradient of 35–90% solvent B over 1 min; 90% B for 1.1 min; and 5% B for 9.9 min [solvent A = 0.1% (v/v) formic acid in water, solvent B = 0.1% (v/v) formic acid in acetonitrile]. Eluted peptides were sprayed into a ThermoFisher linear true quadrupole (LTQ)-FT Ultra mass spectrometer (Thermo Fisher Scientific) using a Bruker-Michrom ADVANCE nanospray source. Survey scans were taken in the Fourier transform (FT, 25000 resolution determined at *m*/*z* 400) and the top 10 ions in each survey scan were then subjected to automatic low energy collision induced dissociation (CID) in the LTQ. The resulting MS/MS spectra were converted to peak lists using MaxQuant2 v1.2.2.5 and searched in MaxQuant with the Andromeda search engine (Cox et al., 2011) against the *Medicago truncatula* v3.5 protein database downloaded from the J. Craig Venter Institute^2^ appended with common contaminants. Results were filtered at 1% False Discovery Rate using a reverse database search. Label-free quantification was also done using MaxQuant. Specific parameters for the search and the analysis are included in the “parameters.txt” and “summary.txt” files in the MaxQuant output file/folder. These and the associated proteomics data files are available at figshare (figshare.com) via this link: http://dx.doi.org/10.6084/m9.figshare.100494. All identified proteins were assigned a CWP number for cataloging purposes that does not represent ranking of any sort.

### *Medicago* annotations and homology searches

Currently, the complete genome sequence of alfalfa is not available. Gene and protein identity between alfalfa and its close relative *M. truncatula* (for which much sequence information is available) are considered to be high. To our knowledge, however, no global comparison between the two species of currently available coding region data has been carried out. Still, comparison of 100 randomly selected alfalfa protein sequences from the National Center for Biotechnology Information (NCBI)^3^ database with their putative *M. truncatula* orthologs shows a median amino acid identity of 98% (see Table S1 in Supplementary Material). Thus, we should be able to identify alfalfa proteins using the *M. truncatula* genome (Young et al., 2011). The identified proteins were exported from the file “proteinGroups.txt” (included in the MaxQuant output folder, Supplementary Material) and organized in Microsoft Excel and Microsoft Access (Microsoft, Redmond, WA, USA). The number in the “id” column in the “proteinGroups.txt” file is included in Table S2 in Supplementary Material for reference. Medicago Gene Index (MGI) names (in the format MedtrXgXXXXXX) were assigned by MaxQuant with the Andromeda search engine. The individual MGI names were linked to the Affymetrix Mt3.5 probe set (Affymetrix, Santa Clara, CA, USA) using the downloaded annotation file on the Affymetrix website^4^. Every probe was linked to the first listed MGI name and the arabidopsis homolog was taken from the Affymetrix GO annotation file MedicagoAnnotateJAO8.xls^5^. All coding region and protein sequences were downloaded^6^. Not all MGI names had a probe representing them in the Affymetrix file: for those a BlastP search versus arabidopsis was carried out http://blast.ncbi.nlm.nih.gov/Blast.cgi (Altschul et al., 1997). *Arabidopsis* homologs identified by either approach were used to compare to proteins identified in previously published cell wall proteomics studies.

### Bioinformatics analysis

For all proteins identified, the subcellular localization was predicted using TargetP^7^ (Emanuelsson et al., 2007). Functional annotation and protein category allocation was done using the InterProScan sequence search^8^ (Apweiler et al., 2001). Annotation of glycoside hydrolases (GHs) and carbohydrate esterases (CEs) was done according to the CAZy database^9^ (Cantarel et al., 2009). Peroxidases were named according to the Peroxibase^10^ (Oliva et al., 2009).

## Results and Discussion

### Alfalfa stem cell wall isolation and protein extraction

The cell wall proteomes were analyzed from two developmental stages of alfalfa stems: less mature, more digestible apical stems, and more mature, less digestible basal stems. Cell wall material from each tissue was isolated by the procedure of Feiz et al. (2006) to minimize cytosolic protein contamination. Although most CWP extraction methods utilize sequential extractions of isolated cell wall material with CaCl_2_ and LiCl, we carried out sequential extractions with EGTA and LiCl. Because Ca^2+^ stabilizes the pectic components of plant cell walls, treatment with the high affinity Ca^2+^ chelator EGTA should help remove these pectic substances and thereby loosen the cell wall matrix to allow better extraction of CWPs (Letham, 1958; Hepler, 2005). Lithium Chloride is thought to, through chaotropic disruptions, separate the wall into an insoluble inner wall layer and a salt-soluble fraction, releasing tightly bound proteins (Hills et al., 1975).

The proteins extracted by EGTA or LiCl from apical or basal stem cell wall materials were resolved by SDS-PAGE for qualitative assessment (Figure 1). The complement of proteins present in the EGTA and LiCl extracts appears to differ substantially in both apical and basal stems. This is in contrast to methods using CaCl_2_ and LiCl extraction where the complement of proteins in the two salt extracts is generally similar (Feiz et al., 2006; Irshad et al., 2008). Surprisingly, the complement of proteins present in apical versus basal stems did not appear to be dramatically different (Figure 1) by SDS-PAGE analysis as might be expected for two tissues that are physiologically and physically so different.

### Proteomic analysis

To investigate which individual proteins were present in the four fractions, they were each analyzed using shotgun proteomics. Peptides generated by in-gel trypsin digestion were identified by reverse-phase UPLC with MS/MS protein identification using the genome of the close alfalfa relative *M. truncatula* (Young et al., 2011) as the reference genome. Spectral counts, a practical, label-free, way to quantify protein abundance in shotgun proteomic studies (Lundgren et al., 2010), of the individual proteins were used to give a rough estimate of protein abundance by analyzing the MS/MS data with MaxQuant (Cox et al., 2011). These relative abundances should be interpreted cautiously, however, as plant material and other resources were not available to replicate the experiment.

Among the four fractions 272 individual proteins were identified (Table 1; Table S2 in Supplementary Material) including 153 proteins not previously identified in cell wall proteomic analyses. The complete dataset of 272 proteins contained 188 proteins (69%) that were predicted by TargetP (Emanuelsson et al., 2007) to be secreted to the exterior of the cell and targeted to the cell wall (Table S2 in Supplementary Material). As reported by Feiz et al. (2006) previous cell wall proteome studies using the traditional high-salt method to isolate cell walls from arabidopsis (Chivasa et al., 2002) or alfalfa (Watson et al., 2004) had only around 50% of the identified proteins predicted to be secreted. Our value of predicted secreted proteins compares favorably with that of Irshad et al., 2008; 79%), who also used the low-salt method in their cell wall isolation. The substantial increase in percentage of proteins predicted to be secreted in our study and that of Irshad et al. (2008) strongly suggests that the low-salt density method improves the quality of the dataset by reducing the level of contaminating intracellular, non-CWPs.

**Table 1:** **Abundant proteins identified in the EGTA and LiCl extracts of cell walls prepared from basal and apical alfalfa stems**.

CWP#^a^	MGI#^b^	MGI-IDs short annotation	TargetP^c^	LFQ values (×10^9^)^d^		
				Basal stem	Apical stem	Total
**Proteins acting on carbohydrates (PAC)**						
#313	Medtr2g034440	GH family 17 (glucan endo-1,3-β-glucosidase)	S	2.36	0.65	3.02
#334	Medtr2g034470	GH family 17 (glucan endo-1,3-β-glucosidase)	S	1.33	0.31	1.63
#324	Medtr8g074330	GH family 19 (endochitinase)	S	1.53	0.41	1.95
#318	Medtr2g094060	GH family 35 (β-galactosidase)	S	0.69	0.99	1.68
#045	Medtr3g118390	GH family 19 (endochitinase)	S	0.86	0.21	1.06
**Oxidoreductases (OR)**						
#322	Medtr8g089110	Blue copper protein, cupredoxin	S	0.68	0.38	1.06
#312	Medtr5g074970	Peroxidase MtPrx29	S	5.37	1.70	7.07
#023	Medtr7g111240	Germin, manganese binding site	S	1.34	0.49	1.83
#333	Medtr3g094650	Peroxidase MtPrx54	S	0.96	0.18	1.15
#315	Medtr3g072190	Peroxidase MtPrx34	S	2.45	1.30	3.74
#007	Medtr2g029820	Peroxidase MtPrx11	Other	2.41	2.10	4.51
#323	Medtr2g084020	Peroxidase MtPrx28	S	0.48	0.96	1.44
#317	Medtr4g095450	Peroxidase MtPrx38	S	0.80	1.01	1.81
#015	Medtr2g029750	Peroxidase MtPrx16	S	0.61	0.13	0.74
#332	Medtr1g085140	Germin-like protein 19	S	1.16	0.58	1.74
**Proteins with interacting domains (ID)**						
#086	Medtr7g023690	Polygalacturonase inhibitor protein, LRR	S	1.42	0.76	2.18
#319	Medtr7g023590	Polygalacturonase inhibitor protein, LRR	S	0.73	2.14	2.87
#083	Medtr7g092740	Polygalacturonase inhibitor protein, LRR	S	0.79	2.00	2.79
#011	Medtr2g098250	Polygalacturonase inhibitor protein, LRR	S	0.07	2.12	2.19
#341	Medtr6g078070	Kunitz-type trypsin inhibitor alpha chain	S	0.38	0.36	0.74
**Proteases (P)**						
#314	Medtr7g081750	Peptidase S8, subtilisin-related	S	3.31	1.18	4.49
**Signaling (S)**						
#140	Medtr4g085480	Receptor protein kinase (LRR)	S	0.53	0.93	1.46
#325	Medtr7g075270	Fasciclin-like arabinogalactan protein 13	S	0.13	0.59	0.73
**Proteins related to lipid metabolism (LM)**						
#321	Medtr4g029350	Lipid-transfer protein and hydrophobic protein	S	1.69	0.93	2.61
#338	Medtr4g050400	Unknown Protein, MD-2-related lipid-recognition	S	0.81	1.10	1.92
**Miscellaneous functions (M)**						
#316	Medtr5g033960	Ubiquitin	Other	0.36	0.41	0.77
#320	Medtr8g014650	Stem 28 kDa glycoprotein, acid phosphatase, plant	S	0.17	1.93	2.10
**Unknown function (UF)**						
#052	Medtr4g094240	Os08g0485000, phosphate-induced protein 1	S	0.61	0.37	0.98

*^a^CWP numbers were assigned arbitrarily and do not represent any ranking*.

^b^*Medicago* MGI# http://gbrowse.jcvi.org/cgi-bin/gbrowse/medicago/

*^c^Prediction of subcellular localization by TargetP (Emanuelsson et al., 2007). S: secreted; other: any other location (i.e., no chloroplast, mitochondria, or secretory pathway signal present)*.

*^d^Label-free Quantitation Intensity (LFQ) as determined by MaxQuant (Cox et al., 2011)*.

*Accession numbers of proteins that were identified for the first time in a cell wall proteome analysis are underlined. Numbers in the “total” column are the Label-free Quantitation Intensity (LFQ) as determined by MaxQuant from the combined EGTA and LiCl fractions for both the apical and basal stems. An LFQ threshold value of 0.73 × 10^9^ was used to select the top 10% most abundant proteins. The complete list of 272 proteins including additional information for each protein is available in Table S2 in Supplementary Material, provided as an Excel file*.

Still, in our study, 31% of the identified proteins are not predicted to be secreted via known mechanisms and are potential intracellular contaminants. Sixteen proteins were predicted to be targeted to the chloroplasts, seven to the mitochondria, and three to the ER. The remaining 58 protein candidates (21% of the total) do not have known targeting sequences and therefore are likely to be intracellular proteins. These might be present in the cell wall preparations due to their biochemical properties causing them to be easily trapped by the cell wall matrix during the isolation procedure. It cannot be ruled out that “non-secreted” proteins are bona fide CWPs that lack known targeting signals and are targeted to the cell wall via some other mechanism.

As no replication was performed, a detailed analysis of individual protein abundance would be difficult, but spectral counts were used to give a rough approximation of abundance. Of the set of 272 identified proteins, 28 proteins represented the approximately 10% most abundant proteins and constituted approximately 71% of the total protein extracted from the cell walls. Among these we identified seven different peroxidases, and three other oxidoreductases (OR), which are likely involved in cross-linking cell wall components such as lignin and protection of the cell from free radicals (Passardi et al., 2004). Also five GHs previously implicated in the biosynthesis and crosslinking of the cellulose and hemicellulose backbones (Cantarel et al., 2009) were highly abundant. Some of the abundant proteins have a role in defense. These include four leucine-rich-repeat (LRR) domain containing polygalacturonase inhibitor proteins, which have been shown to defend against pathogen induced breakdown of the cell wall (De Lorenzo et al., 2001) and a LRR domain containing receptor kinase that has been suggested to be crucial for host-pathogen interaction (Decreux and Messiaen, 2005). One lipid-transfer proteins, a serine peptidase, and a fasciclin-like arabinogalactan protein (AGP) were also present in this group of high abundant proteins and have been associated with the maturation of the cell wall and secondary growth (Johnson et al., 2003; Nieuwland et al., 2005; Vartapetian et al., 2011). The list is completed with an acid phosphatase glycoprotein, ubiquitin, a trypsin inhibitor, and two proteins of unknown function (UF), one containing a lipid-recognition domain (Table 1). Whereas 22 of the most abundant proteins identified here were previously identified in cell wall proteomic studies, the remaining six are newly identified as part of a cell wall proteome. The identification of new high abundance proteins might be due to the less biased nature of the shotgun approach used here. However, the bias in older approaches would be expected to favor abundant proteins since these might be more likely to be manually selected from 2-D gels for sequencing. Thus, an alternative explanation is that these proteins are unique to alfalfa or to the more mature tissues used here.

In other studies, CaCl_2_ and LiCl salt extraction fractions have tended to be similar and were sometimes pooled before analysis (Watson et al., 2004; Irshad et al., 2008). Here, the EGTA and LiCl fractions were analyzed separately and, based on the proteins identified are quite distinct as was expected from the SDS-PAGE analysis (Figure 1). The EGTA fraction contained 37 unique proteins (i.e., not present in the LiCl fraction), of which 25 proteins were not identified previously in a cell wall proteome. The LiCl fraction contained 63 unique proteins, with 46 of those being identified for the first time as CWPs. The remaining 172 proteins were found in both fractions, but, based on spectral count data many may be more prevalent in one fraction than the other (Table S2 in Supplementary Material). To our knowledge, ours is the first cell wall proteomic study to utilize EGTA to extract proteins. Our findings suggest that the actions of EGTA and LiCl in protein extraction from the cell walls are quite distinct, given the relatively low level of overlap between the two fractions, but the biological significance of this is at present unclear. Proteins requiring LiCl for solubilization may be more strongly associated with the wall matrix and/or may be more deeply sequestered in the matrix, perhaps by being deposited earlier and hence more difficult to solubilize.

Between the two stem maturities, based on the proteins identified there is a relatively large overlap of the cell wall proteome consistent with the SDS-PAGE analysis (Figure 1). The basal section of the stems contained only 12 unique proteins (i.e., not present in the apical stems) of which seven have not been previously identified as CWPs. The apical sections of stems contained 19 unique proteins, with 14 newly identified as being associated with the cell wall. The remaining 245 proteins were found in both fractions, although in some cases particular proteins appear to be more prevalent in one fraction than the other.

### Functional classification of proteins from alfalfa stem cell walls

**Figure 2:**
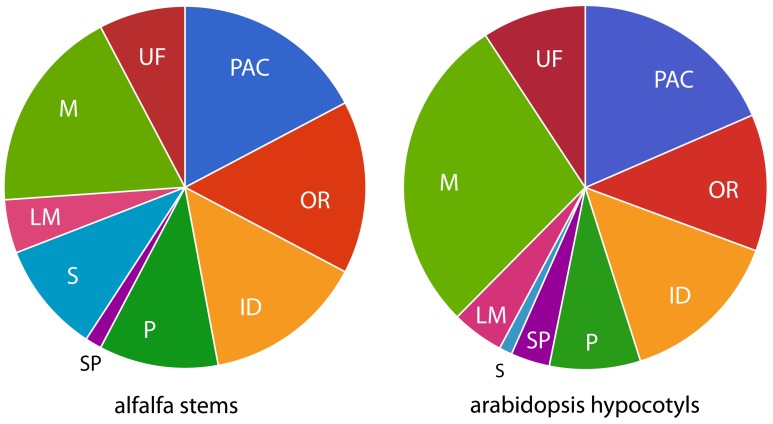
**Distribution of functional classes from proteomic studies**. Functional classes are: PAC, proteins acting on carbohydrates; OR, oxidoreductases; ID, proteins with interacting domains; P, proteases; S, signaling; LM, proteins related to lipid metabolism; M, miscellaneous functions; UF, unknown function.

To gain additional insight into the roles played by the identified proteins in the cell well, they were clustered based on their predicted biochemical functions using the classes defined by Jamet et al. (2006). Those functional classes are: proteins acting on carbohydrates (PAC), OR, proteins with interacting domains (ID), proteases (P), SPs, proteins involved in signaling (S), proteins related to lipid metabolism (LM), proteins with miscellaneous functions (M), and proteins with UF. Table 2 and Figure 2 show the distribution of the 272 identified proteins by class. Because a distribution of all the identified proteins based simply on presence in the data set might not give an accurate picture of the relative importance of the functional classes, we also used spectral counts to make a rough estimate of the protein abundance in each functional class. This is important since, for example, a particular functional class could have a low number of high abundance proteins, or a high number of low abundance proteins. This latter scenario is the case for the M class, that has many members (50, 18% of the 272 identified proteins), but as a whole constitutes only a relatively small fraction (6%) of CWP extracted. In contrast, PAC, OR, and ID classes both have many members and constitute a relatively large fraction of the CWP extracted (16, 35, and 16%, respectively). All the other functional classes each constituted less than 10% of the extracted CWP, with SP being the least abundant class.

**Table 2:** **Distribution of identified proteins and amount of protein from alfalfa stem cell walls by functional class**.

Functional class	Proteins identified		Fraction of cell wall protein
	#	%	%
PAC	47	17	16
OR	42	15	35
ID	39	14	16
P	29	11	9
SP	4	1	1
S	27	10	7
LM	13	5	7
M	50	18	6
UF	21	8	3
Total	272	100	100

*PAC, proteins acting on carbohydrates; OR, oxidoreductases; ID, proteins with interacting domains; P, proteases; S, signaling; LM, proteins related to lipid metabolism; M, miscellaneous functions; UF, unknown function*.

A large number of glycoside hydrolase family proteins were identified in the PAC category, most prevalent being endo-beta-1,3-glucanases (GH family 17) and beta-galactosidases (GH family 35), which are both involved in the processing of cell wall matrix carbohydrates (Cantarel et al., 2009). Twenty-two of the proteins in the OR class were peroxidases (Table 1). Peroxidases are involved in the crosslinking of phenolic compounds, including lignin (Passardi et al., 2004), and would be expected to play an important role in the physiology of the cell wall. We identified a higher number of peroxidases (22 versus 3) than were identified in a previous proteomic study of alfalfa stems (Watson et al., 2004). This may be mostly a reflection of the larger number of proteins identified in our study, since the fractions of peroxidases among identified proteins (8% here versus 5% in Watson et al., 2004) were similar between the two studies.

Only four SP class proteins were identified, and these did not appear to be particularly abundant based on spectral count data. This low number of SP in the dataset could be due to incorrect annotation as such SPs do not always have clear domains that can be used for classifications (Cassab, 1998). Low recovery of SPs could also be caused by the use of material that, even for the apical stems, was too mature to allow the SPs to be extracted from the cell wall. Even younger tissues (e.g., the most apical one or two internodes) might be a source of SPs that are not yet covalently bound, but so far studies of undifferentiated cell cultures and young hypocotyls have not resulted in identification of substantially more SPs [e.g., only six SP’s were identified from 5- to 11-day-old hypocotyls (Irshad et al., 2008)]. Thus, new methodologies and approaches may be required to identify additional, strongly bound SPs. For example, bioinformatics tools, such as prediction of localization or more advanced domain searches, could be used to identify other SPs from genome sequence data. Alternatively, a biochemical approach might be treatment of cell wall residues, which we know still contain proteins even after extraction with EGTA and LiCl (data not shown), with protein cleavage reagents (e.g., proteases or chemicals). This might allow release of peptides from proteins covalently bound to the cell wall matrix so that they could be sequenced and allow the corresponding proteins to be identified.

### Cross study comparison of cell wall proteins

To assist in analyzing our data in the context of other studies, we created a database of proteins identified in 18 different cell wall proteomic studies (Table S3 in Supplementary Material). Although most cell wall proteome analyses have been carried out using various arabidopsis tissues, our database also included data from chickpea seedlings, tobacco suspension culture and leaves, and alfalfa stems. For proteins of these non-arabidopsis species, the arabidopsis homologs were identified to facilitate matching of proteins across species.

When looking at the complete database, there is relatively little overlap in the identified proteins among the different studies, including ours. This may be due to different species and tissues being used in these proteomic studies. Methodological differences may also play a large role in the lack of overlap. For example, most cell wall proteome studies have utilized sequencing of spots isolated from 2-D gels, which may tend to be biased toward more abundant proteins.

Given the similar approach with respect to isolating cell walls and use of shotgun proteomics, the most relevant comparison might be our study to a recent study by Irshad et al. (2008) of arabidopsis hypocotyls (173 proteins identified). Sixteen of the proteins of this arabidopsis study corresponded to 37 alfalfa proteins (there are instances of multiple distinct alfalfa proteins being homologous to a single arabidopsis protein, suggesting larger gene families in tetraploid alfalfa). But the larger portion of the proteins identified, 78 in arabidopsis and 229 in alfalfa, are unique to their respective datasets. Still the distribution of functional categories over alfalfa and arabidopsis datasets is similar (Figure 2), suggesting a different proteome (i.e., not a homologous one) carries out similar functions in the cell walls of the two tissues from which they are derived. This could reflect species differences, tissue differences, or both. Between the two datasets, most proteins are in the PAC, OR, ID, P, and M classes, and lower proportions in LM and SP classes. The most striking difference is in category S, which is much larger in alfalfa, possibly due to the differences in growing conditions of the plants used in these two studies. The arabidopsis plants used in Irshad et al. (2008) were grown under sterile climate-controlled conditions and harvested when the plants were less than 11 days old (Irshad et al., 2008). It might be that the considerably older alfalfa stems, grown outside exposed to variable environmental conditions might contain more proteins that are involved in signaling. Proportions in M and UF classes also appear to differ between the arabidopsis study and ours, but as described above, this may be a reflection of the artificial nature of these classes.

## Conclusion

We have carried out a cell wall proteomic study on two maturities of the stems of alfalfa, an important forage crop. We used a modified CWP extraction procedure utilizing EGTA to remove pectins, followed by a LiCl extraction to isolate more tightly bound proteins. Surprisingly no clear difference in the proteomes of the two maturities of alfalfa stems examined was seen even though there is usually a substantial decrease in digestibility of alfalfa stems with maturity. This may indicate that future studies should focus on tissues even more divergent with respect to maturity than were used here, or that different experimental approaches may be required (e.g., important proteins may become cross-linked into the cell wall matrix at later maturity and avoid detection by standard methods). In analyzing available CWP data to date, we found there are surprisingly few individual proteins common among multiple cell wall proteome studies, likely due to differences in tissues used and experimental approach employed. Continuing cell wall proteome studies using newly available shotgun methodologies will undoubtedly provide additional insight into the basic physiology of plant cell wall development. Further, this could assist in the longer term goal of identifying CWP targets for modification, either by conventional selection or genetic engineering, in crop plants that will lead to more desirable cell wall properties.

## Conflict of Interest Statement

The authors declare that the research was conducted in the absence of any commercial or financial relationships that could be construed as a potential conflict of interest.

## Supplementary Material

The Supplementary Material for this article can be found online at http://www.frontiersin.org/Plant_Proteomics/10.3389/fpls.2012.00279/abstract

## Footnotes

^1^http://rtsf.msu.edu/proteomics.html

^2^www.jcvi.org

^3^http://www.ncbi.nlm.nih.gov/

^4^http://www.affymetrix.com/browse/products.jsp?productId=131472&navMode=34000&navAction=jump&aId=productsNav#1_3, filename: Mt3.5V3_Illumina_08302010.probe2genes.map

^5^http://www.medicago.org/GeneChip/

^6^http://www.jcvi.org/cgi-bin/medicago/download.cgi

^7^http://www.cbs.dtu.dk/services/TargetP/

^8^http://www.ebi.ac.uk/Tools/pfa/iprscan/

^9^http://www.cazy.org/

^10^http://peroxibase.toulouse.inra.fr/

## References

[B1] Altschul S. F. Madden T. L. Schaffer A. A. Zhang J. Zhang Z. Miller W. (1997). Gapped BLAST and PSI-BLAST: a new generation of protein database search programs. Nucleic Acids Res. 25, 3389–3402 10.1093/nar/25.17.3389 9254694 PMC146917

[B2] Apweiler R. Attwood T. K. Bairoch A. Bateman A. Birney E. Biswas M. (2001). The InterPro database, an integrated documentation resource for protein families, domains and functional sites. Nucleic Acids Res. 29, 37–40 10.1093/nar/29.7.e37 11125043 PMC29841

[B3] Bayer E. M. Bottrill A. R. Walshaw J. Vigouroux M. Naldrett M. J. Thomas C. L. (2006). Arabidopsis cell wall proteome defined using multidimensional protein identification technology. Proteomics 6, 301–311 10.1002/pmic.200500046 16287169

[B4] Bhushan D. Pandey A. Chattopadhyay A. Choudhary M. K. Chakraborty S. Datta A. (2006). Extracellular matrix proteome of chickpea (Cicerarietinum L.) illustrates pathway abundance, novel protein functions and evolutionary perspect. J. Proteome Res. 5, 1711–1720 10.1021/pr060116f 16823979

[B5] Borderies G. Jamet E. Lafitte C. Rossignol M. Jauneau A. Boudart G. (2003). Proteomics of loosely bound cell wall proteins of Arabidopsis thaliana cell suspension cultures: a critical analysis. Electrophoresis 24, 3421–3432 10.1002/elps.200305608 14595688

[B6] Borner G. H. Lilley K. S. Stevens T. J. Dupree P. (2003). Identification of glycosylphosphatidylinositol-anchored proteins in Arabidopsis. A proteomic and genomic analysis. Plant Physiol. 132, 568–577 10.1104/pp.103.021170 12805588 PMC166998

[B7] Boudart G. Jamet E. Rossignol M. Lafitte C. Borderies G. Jauneau A. (2005). Cell wall proteins in apoplastic fluids of Arabidopsis thaliana rosettes: identification by mass spectrometry and bioinformatics. Proteomics 5, 212–221 10.1002/pmic.200400882 15593128

[B8] Brady J. D. Sadler I. H. Fry S. C. (1996). Di-isodityrosine, a novel tetrametric derivative of tyrosine in plant cell wall proteins: a new potential cross-link. Biochem. J. 315(Pt 1), 323–327 8670125 10.1042/bj3150323 PMC1217189

[B9] Buchanan B. Gruissem W. Jones R. (2000). Biochemistry and Molecular Biology of Plants, 1st Edn. Rockville, MD: American Society of Plant Physiologists

[B10] Burke D. Kaufman P. McNeil M. Albersheim P. (1974). The structure of plant cell walls: VI. A survey of the walls of suspension-cultured monocots. Plant Physiol. 54, 109–115 10.1104/pp.54.1.109 16658824 PMC541512

[B11] Cantarel B. Coutinho P. Rancurel C. Bernard T. Lombard V. Henrissat B. (2009). The Carbohydrate-Active EnZymes database (CAZy): an expert resource for glycogenomics. Nucleic Acids Res. 37, D233–D238 10.1093/nar/gkn663 18838391 PMC2686590

[B12] Cassab G. Varner J. (1988). Cell-wall proteins. Annu. Rev. Plant Physiol. Plant Mol. Biol. 39, 321–353 10.1146/annurev.pp.39.060188.001541 15012236

[B13] Cassab G. I. (1998). Plant cell wall proteins. Annu. Rev. Plant Physiol. Plant Mol. Biol. 49, 281–309 10.1146/annurev.arplant.49.1.281 15012236

[B14] Charmont S. Jamet E. Pont-Lezica R. Canut H. (2005). Proteomic analysis of secreted proteins from Arabidopsis thaliana seedlings: improved recovery following removal of phenolic compounds. Phytochemistry 66, 453–461 10.1016/j.phytochem.2004.12.013 15694452

[B15] Chivasa S. Ndimba B. K. Simon W. J. Robertson D. Yu X. L. Knox J. P. (2002). Proteomic analysis of the Arabidopsis thaliana cell wall. Electrophoresis 23, 1754–1765 10.1002/1522-2683(200206)23:11<1754::AID-ELPS1754>3.0.CO;2-E 12179997

[B16] Cox J. Neuhauser N. Michalski A. Scheltema R. A. Olsen J. V. Mann M. (2011). Andromeda: a peptide search engine integrated into the MaxQuant environment. J. Proteome Res. 10, 1794–1805 10.1021/pr101065j 21254760

[B17] Dani V. Simon W. J. Duranti M. Croy R. R. (2005). Changes in the tobacco leaf apoplast proteome in response to salt stress. Proteomics 5, 737–745 10.1002/pmic.200401119 15682462

[B18] De Lorenzo G. D’Ovidio R. Cervone F. (2001). The role of polygalacturonase-inhibiting proteins (PGIPs) in defense against pathogenic fungi. Annu. Rev. Phytopathol. 39, 313–335 10.1146/annurev.phyto.39.1.313 11701868

[B19] Decreux A. Messiaen J. (2005). Wall-associated kinase WAK1 interacts with cell wall pectins in a calcium-induced conformation. Plant Cell Physiol. 46, 268–278 10.1093/pcp/pci026 15769808

[B20] Emanuelsson O. Brunak S. Von Heijne G. Nielsen H. (2007). Locating proteins in the cell using TargetP, SignalP and related tools. Nat. Protoc. 2, 953–971 10.1038/nprot.2007.131 17446895

[B21] Feiz L. (2004). Cell Wall Proteomics of Elongating Tissues of Arabidopsis thaliana. M.Sc thesis, Université Paul Sabatier, France, 28

[B22] Feiz L. Irshad M. Pont-Lezica R. F. Canut H. Jamet E. (2006). Evaluation of cell wall preparations for proteomics: a new procedure for purifying cell walls from Arabidopsis hypocotyls. Plant Methods 2, 10 10.1186/1746-4811-2-10 16729891 PMC1524762

[B23] Grabber J. H. Panciera M. T. Hatfield R. D. (2002). Chemical composition and enzymatic degradability of xylem and nonxylem walls isolated from alfalfa internodes. J. Agric. Food Chem. 50, 2595–2600 10.1021/jf011598c 11958628

[B24] Griffin N. M. Yu J. Long F. Oh P. Shore S. Li Y. (2010). Label-free, normalized quantification of complex mass spectrometry data for proteomic analysis. Nat. Biotechnol. 28, 83–89 10.1038/nbt.1592 20010810 PMC2805705

[B25] Hatfield R. (1992). Carbohydrate-composition of alfalfa cell-walls isolated from stem sections differing in maturity. J. Agric. Food Chem. 40, 424–430 10.1021/jf00015a012

[B26] Hepler P. (2005). Calcium: a central regulator of plant growth and development. Plant Cell 17, 2142–2155 10.1105/tpc.105.032508 16061961 PMC1182479

[B27] Hills G. Phillips J. Gay M. Roberts K. (1975). Self-assembly of a plant-cell wall in vitro. J. Mol. Biol. 96, 431–441 10.1016/0022-2836(75)90170-9 1100848

[B28] Irshad M. Canut H. Borderies G. Pont-Lezica R. Jamet E. (2008). A new picture of cell wall protein dynamics in elongating cells of Arabidopsis thaliana: confirmed actors and newcomers. BMC Plant Biol. 8:94 10.1186/1471-2229-8-94 18796151 PMC2551616

[B29] Jamet E. Albenne C. Boudart G. Irshad M. Canut H. Pont-Lezica R. (2008). Recent advances in plant cell wall proteomics. Proteomics 8, 893–908 10.1002/pmic.200700938 18210371

[B30] Jamet E. Canut H. Boudart G. Pont-Lezica R. F. (2006). Cell wall proteins: a new insight through proteomics. Trends Plant Sci. 11, 33–39 10.1016/j.tplants.2005.11.006 16356755

[B31] Johnson K. L. Jones B. J. Bacic A. Schultz C. J. (2003). The fasciclin-like arabinogalactan proteins of Arabidopsis. A multigene family of putative cell adhesion molecules. Plant Physiol. 133, 1911–1925 10.1104/pp.103.031237 14645732 PMC300743

[B32] Kwon H. K. Yokoyama R. Nishitani K. (2005). A proteomic approach to apoplastic proteins involved in cell wall regeneration in protoplasts of Arabidopsis suspension-cultured cells. Plant Cell Physiol. 46, 843–857 10.1093/pcp/pci089 15769804

[B33] Laemmli U. K. (1970). Cleavage of structural proteins during the assembly of the head of bacteriophage T4. Nature 227, 680–685 10.1038/227680a0 5432063

[B34] Letham D. S. (1958). Maceration of plant-tissues with ethylene-diamine-tetra-acetic acid. Nature 181, 135–136 10.1038/181135a0

[B35] Lundgren D. H. Hwang S. I. Wu L. Han D. K. (2010). Role of spectral counting in quantitative proteomics. Expert Rev. Proteomics 7, 39–53 10.1586/epr.09.69 20121475

[B36] Melan M. A. Cosgrove D. J. (1988). Evidence against the involvement of ionically bound cell wall proteins in pea epicotyl growth. Plant Physiol. 86, 469–474 10.1104/pp.86.2.469 11538235 PMC1054508

[B37] Millar D. J. Whitelegge J. P. Bindschedler L. V. Rayon C. Boudet A. M. Rossignol M. (2009). The cell wall and secretory proteome of a tobacco cell line synthesising secondary wall. Proteomics 9, 2355–2372 10.1002/pmic.200800721 19402043

[B38] Minic Z. Jamet E. Negroni L. Arsene Der Garabedian P. Zivy M. Jouanin L. (2007). A sub-proteome of Arabidopsis thaliana mature stems trapped on Concanavalin A is enriched in cell wall glycoside hydrolases. J. Exp. Bot. 58, 2503–2512 10.1093/jxb/erm082 17526915 PMC2394711

[B39] Minic Z. Jamet E. San-Clemente H. Pelletier S. Renou J. Rihouey C. (2009). Transcriptomic analysis of Arabidopsis developing stems: a close-up on cell wall genes. BMC Plant Biol. 9:6 10.1186/1471-2229-9-6 19149885 PMC2649120

[B40] Morrow D. Jones R. (1986). Localization and partial characterization of the extracellular proteins centrifuged from pea internodes. Physiol. Plant. 67, 397–407 10.1111/j.1399-3054.1986.tb05754.x

[B41] Nesvizhskii A. I. Keller A. Kolker E. Aebersold R. (2003). A statistical model for identifying proteins by tandem mass spectrometry. Anal. Chem. 75, 4646–4658 10.1021/ac0341261 14632076

[B42] Nieuwland J. Feron R. Huisman B. A. Fasolino A. Hilbers C. W. Derksen J. (2005). Lipid transfer proteins enhance cell wall extension in tobacco. Plant Cell 17, 2009–2019 10.1105/tpc.105.032094 15937228 PMC1167548

[B43] Oliva M. Theiler G. Zamocky M. Koua D. Margis-Pinheiro M. Passardi F. (2009). PeroxiBase: a powerful tool to collect and analyse peroxidase sequences from Viridiplantae. J. Exp. Bot. 60, 453–459 10.1093/jxb/ern317 19112168

[B44] Passardi F. Penel C. Dunand C. (2004). Performing the paradoxical: how plant peroxidases modify the cell wall. Trends Plant Sci. 9, 534–540 10.1016/j.tplants.2004.09.002 15501178

[B45] Robertson D. Mitchell G. P. Gilroy J. S. Gerrish C. Bolwell G. P. Slabas A. R. (1997). Differential extraction and protein sequencing reveals major differences in patterns of primary cell wall proteins from plants. J. Biol. Chem. 272, 15841–15848 10.1074/jbc.272.17.11336 9188482

[B46] Schultz C. J. Ferguson K. L. Lahnstein J. Bacic A. (2004). Post-translational modifications of arabinogalactan-peptides of Arabidopsis thaliana. Endoplasmic reticulum and glycosylphosphatidylinositol-anchor signal cleavage sites and hydroxylation of proline. J. Biol. Chem. 279, 45503–45511 10.1074/jbc.M313017200 15322080

[B47] Shevchenko A. Wilm M. Vorm O. Mann M. (1996). Mass spectrometric sequencing of proteins silver-stained polyacrylamide gels. Anal. Chem. 68, 850–858 10.1021/ac950914h 8779443

[B48] Vanholme R. Morreel K. Ralph J. Boerjan W. (2008). Lignin engineering. Curr. Opin. Plant Biol. 11, 278–285 10.1016/j.pbi.2008.03.005 18434238

[B49] Vartapetian A. B. Tuzhikov A. I. Chichkova N. V. Taliansky M. Wolpert T. J. (2011). A plant alternative to animal caspases: subtilisin-like proteases. Cell Death Differ. 18, 1289–1297 10.1038/cdd.2011.49 21546909 PMC3172098

[B50] Watson B. S. Lei Z. Dixon R. A. Sumner L. W. (2004). Proteomics of Medicago sativa cell walls. Phytochemistry 65, 1709–1720 10.1016/j.phytochem.2004.04.026 15276432

[B51] Wessel D. Flugge U. I. (1984). A method for the quantitative recovery of protein in dilute solution in the presence of detergents and lipids. Anal. Biochem. 138, 141–143 10.1016/0003-2697(84)90782-6 6731838

[B52] Yang S. S. Xu W. W. Tesfaye M. Lamb J. F. Jung H. J. Vandenbosch K. A. (2010). Transcript profiling of two alfalfa genotypes with contrasting cell wall composition in stems using a cross-species platform: optimizing analysis by masking biased probes. BMC Genomics 11:323 10.1186/1471-2164-11-323 20497574 PMC2893600

[B53] Young N. D. Debelle F. Oldroyd G. E. Geurts R. Cannon S. B. Udvardi M. K. (2011). The Medicago genome provides insight into the evolution of rhizobial symbioses. Nature 480, 520–524 10.1038/480162a 22089132 PMC3272368

[B54] Zhu J. Alvarez S. Marsh E. L. Lenoble M. E. Cho I. J. Sivaguru M. (2007). Cell wall proteome in the maize primary root elongation zone. II. Region-specific changes in water soluble and lightly ionically bound proteins under water deficit. Plant Physiol. 145, 1533–1548 10.1104/pp.107.103713 17951457 PMC2151692

